# Advances in Yeast Probiotic Production and Formulation for Preventative Health

**DOI:** 10.3390/microorganisms12112233

**Published:** 2024-11-04

**Authors:** Ghaneshree Moonsamy, Yrielle Roets-Dlamini, Cebeni Nkosihawukile Langa, Santosh Omrajah Ramchuran

**Affiliations:** Council for Scientific and Industrial Research (CSIR) Future Production Chemicals, Meiring Naude Drive, Pretoria 0081, South Africa; yroets@csir.co.za (Y.R.-D.); clanga@csir.co.za (C.N.L.); sramchuran@csir.co.za (S.O.R.)

**Keywords:** probiotics, yeast, biotherapeutics, formulation

## Abstract

The use of probiotics has been gaining popularity in terms of inclusion into human diets over recent years. Based on properties exerted by these organisms, several benefits have been elucidated and conferred to the host. Bacteria have been more commonly used in probiotic preparations compared to yeast candidates; however, yeast exhibit several beneficial properties, such as the prevention and treatment of diarrhea, the production of antimicrobial agents, the prevention of pathogen adherence to intestinal sites, the maintenance of microbial balance, the modulation of the immune system, antibiotic resistance, amongst others. *Saccharomyces boulardii* is by far the most studied strain; however, the potential for the use of other yeast candidates, such as *Kluyveromyces lactis* and *Debaryomyces hansenii*, amongst others, have also been evaluated in this review. Furthermore, a special focus has been made regarding the production considerations for yeast-based probiotics and their formulation into different delivery formats. When drafting this review, evidence suggests that the use of yeasts, both wild-type and genetically modified candidates, can extend beyond gut health to support skin, the respiratory system, and overall immune health. Hence, this review explores the potential of yeast probiotics as a safe, effective strategy for preventative health in humans, highlighting their mechanisms of action, clinical applications, and production considerations.

## 1. Introduction

Probiotics and their use for human health implications have been studied extensively, and in more recent years, their acceptance for use by the global population has seen a positive trajectory. According to the most widely accepted definition, a probiotic is known to be “live microorganisms which when administered in adequate amounts confer a health benefit on the host” [1].

Generally, only bacterial and yeast organisms have been classified as probiotics, with the most common being Lactobacilli, Bifidobacteria, Enterococci, Faecalibacterium, Clostridia, and more recently Propionibacteria [2]. Upon application, these organisms have been found to prevent and treat various clinical diseases, improve the intestinal micro-environment, prevent physiological stress and the proliferation of pathogens, improve the health of the intestinal epithelium, modulate immunological homeostasis, amongst others [3,4]. Historically, negative perceptions plagued yeast cultures, as these organisms were generally referred to as pathogenic, disease-causing microbes. However, several studies centered on the use of non-pathogenic yeasts that possess probiotic properties have emerged, which highlights their innate ability to influence physiology, metabolism, and immune homeostasis in the colon [5]. Yeasts have been studied and have proven to be effective starter cultures, and significant interest has been noted in their use in various biotechnological applications [6].

Yeasts make up <0.1% of the human microbiome. Most yeast isolates that have been isolated from the human microbiome include *Candida albicans*, *Torulopsis glabratra*, *Candida tropicalis*, *Malassezia* spp., and *Saccharomyces* spp. [4,7]. Other probiotic yeast candidates include *Cryptococcus* spp. [8], *Candida famata* [9], *C. tropicalis* [10], *Debaryomyces hansenii* [11], *Issatchenkia orientalis* [10], *Kluyveromyces lactis* [12], *Kluyveromyces marxianus* [12,13,14], *Metschnikowia gruessii* [13], *Pichia jadinii*, *Pichia kluyveri* [10], *Pichia kudriavzevii* [10], *Pichia pastoris* [15], *Pichia guilliermondii* [16], and *Wickeramomyces anomalus* [16]. The ability to resist low pHs, the presence of digestive enzymes, bile salts, and organic acids make these organisms ideal candidates to serve as probiotics [7].

Yeasts that have been classified as generally regarded as safe (GRAS) have been shown to have several health implications on the human host. These influences may include but are not limited to being effective on gut microbiota dysbiosis and possess anti-inflammatory, anti-proliferative, anti-cancer and anti-allergenic properties [5,17].

*Saccharomyces boulardii*, *Saccharomyces cerevisiae*, and *Candida* spp. are the most common yeasts used as probiotics, and are used most for the treatment of *Clostridium difficile* diarrhea [4]. *S. boulardii* was first isolated from litchis in Indochina and is not autochthonous in the microbiome [7]. However, this non-pathogenic yeast, amongst other species such as *Saccharomyces cerevisiae*, *Pichia kudriavzevii*, *Candida famata*, *Kluyveromyces lactis*, *Debaryomyces hansenii*, and *Issatchenkia orientalis*, have been found to confer the following probiotic effects to the host, advocating their use for health benefits (Table 1).

Based on the eight probiotics candidate evaluated in Table 1, the probiotic effects ranged from antimicrobial activity to gut flora restoration, immune modulation and glycemic control, antioxidant properties, cholesterol reduction, bile salt resistance, adherence to the gut lining, pathogen inhibition, and biofilm formations (Table 1). Additionally, the impact of including probiotics, both wild-type and genetically modified probiotics (yeast and bacterial), are detailed in Table 2. Findings range from using probiotics to enhance immune function, the alleviation of mental health issues, supporting cardiovascular health, improving metabolic function as well as preventing allergies, and contributing to oral health (Table 2).

Based on the numerous applications detailed in Table 3, specific characteristics for probiotic candidate selections must be applied in order to select an appropriate candidate or consortium of organisms. According to Arevalo-Villena et al. [6], when developing a yeast probiotic product, the following characteristics ought to be sought (Table 1). Other factors that may be considered include the assimilation of cholesterol; the deconjugation of bile salts; the demonstration of antioxidant, hemolytic, cytotoxicity, and the ability to produce cytokines and phytase.

## 2. Methodology

This review provided a narrative that focused on the potential of using yeast as a probiotic and was performed by (i) searching for relevant literature sources; (ii) reviewing the associated content; and (iii) discussing the findings. There is a large body of knowledge that details the use of bacterial organisms as probiotics, with successful applications; however, limited reviews specific to the advantages of using yeast candidates have been published. Several databases, such as PubMed, Scopus, Web of Science, Science Direct, and Google Scholar, were used to identify relevant studies. The final search was conducted in August 2024 using the following keywords: probiotics, yeast, biotherapeutics, and formulation. All relevant sources that were identified after the search was complete were evaluated for suitability, and these sources were summarized and synthesized for inclusion into this review.

## 3. Status of the Biomanufacturing of Yeast Probiotics

Yeast biomass production is a common practice across the globe [43]. During probiotic production, for the interest of this review on yeast probiotics, the primary focus of process optimization is to maximize volumetric organism productivity [44].

### 3.1. Key Bioprocess Considerations

The main contributing factors to this key process indicator are composition and components present in the growth medium; operational parameters used in the process, such as temperature, pH, and aeration; and mode of cultivations, either batch, fed-batch, or continuous.

**Figure 1:**
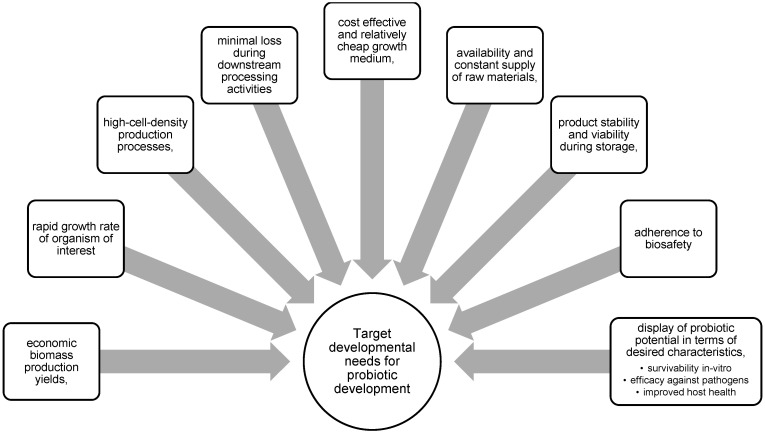
Factors to consider when developing a yeast probiotic using a classical biotechnological approach.

Despite there being several commercial probiotics available, the focus of producers is to ensure that these products can be produced economically to increase market share [44]. At present, major producers of probiotics have to date developed highly efficient, refined, and vertically integrated microbial production systems [44]. Upon biomanufacturing of probiotics, the following characteristics are of vital importance to promote the uptake of the technology (Figure 1).

Probiotics are typically produced in stirred tank bioreactors, and growth conditions must be optimized per strain to achieve high-cell-density cultivations [45]. Different strategies can be evaluated to maximize the quantity of cells produced per batch in the shortest period, attributing to the productivity of the process. High-cell-density cultivations (HCDCs) of microorganisms can have varying outputs depending on the type of organism being cultivated [46]. For yeasts, HCDC biomass concentrations of ~170 g·L^−1^ are acceptable, whereas for Lactobacillus species, a concentration of ~63 to 118 g·L^−1^ meets this threshold. Reports by Kamoshita et al. [47] described achieving biomass concentrations of ~140 g·L^−1^ using an HCDC process for *Lactobacillus lactis*, making it one of the highest thresholds for probiotic organisms, achieved to date. Medium formulations and the availability of raw materials are critical factors when designing HCDC cultivation processes.

There are several strategies that can be applied to achieve HCDC cultivations, and researchers skilled in the art of bioprocess development would have individual approaches and know-how to achieve this. When developing HCDCs, it is imperative to set target performance thresholds for production. For Bacillus cultivations, thresholds of ~1 × 10^10^ spores per mL are targeted to minimize production costs and to also allow for potential losses of cells during downstream production activities [48]. For some probiotic organisms, such as a microaerophilic *Lactobacillus reuteri* strain, these targets of 1 × 10^10^ cells per mL are difficult to achieve despite attempting several process interventions to boost productivity.

Strain selection is critical in ensuring that HCDCs can be achieved. Some organisms, despite having superlative properties as suitable probiotic, cannot be produced in sufficient quantities in bioreactors [49]. Strategies that could be evaluated include the mode of cultivation used for production that is using either batch, fed-batch, or continuous operations. Batch cultivations result in shorter process times; however, they have limitations in terms of the maximum amounts of cells achievable in the bioreactor, using nutrients and supplements provided in the initial charge medium. Fed-batch processes circumvent some of the limitations of batch cultivation processes, as feeding regimes can be applied to maximize biomass production, resulting in higher productivity than batch processes. Continuous cultivation processes are seldom used for probiotic production, as fed-batch production technologies generally outweigh the continuous process in terms of yield and productivity [50]. Perfusion-based cultivations are generally not suitable for probiotic cultivations as they are more tailored for the production of extracellular metabolites [51].

As these probiotic preparations are being manufactured for human consumption, it is imperative to ensure that the manufacturing process is validated at the manufacturing scale to ensure batch-to-batch consistency [52]. Formulation technology for probiotics, predominantly maintaining product viability while minimizing losses, is generally proprietary to industry; however, the stability and shelf-life of the product are key quality attributes that must be maintained [49]. Depending on whether the probiotic product is being used for drug (medical benefit) or dietary supplements, regulatory requirements differ. For consumers, information pertaining to the biosafety level of the organism used in the product, safe delivery, and functionality must be made available to empower users to select the most suitable product for use [53].

### 3.2. Advances in Yeast Probiotic Manufacture

Conventional yeast production requires a cultivation medium that typically contains carbon, nitrogen, vitamins, and trace metals [54]. As the growth medium for probiotic production is a major consideration in process development, other non-conventional fermentation feedstocks are being considered to produce probiotics to minimize production costs (Table 2). Agro-industrial residues, such as molasses, have been used for the cultivation of yeast probiotics [17]. Molasses is a viscous, sugar-rich nutrient source that contains ~34% sucrose, glucose, fructose, and other minerals. In sugar-producing countries, such as Brazil and South Africa, ~10 million tons of sugar cane molasses is discharged [17], hence making it a suitable nutrient feedstock for the large-scale production and manufacturing of probiotics.

Additionally, with the rise in food production, food waste volumes are also on the increase. Hence, the food waste that is accumulated is rich in proteins, carbohydrates, and lipids, and therefore, it can be used as a suitable substrate to cultivate microorganisms [55]. This circular economy, in some instances referred to as bioeconomy initiatives, is intended to reduce the economic, societal, and environmental costs and to drive waste to wealth activities [55]. Probiotics are not usually the intended product produced using waste valorization initiatives; however, there have been some successful attempts in demonstrating the concept in studies conducted by Patil et al. [25] and Kitamura et al. [17]. These studies demonstrated the production of *Kluyveromyces*, *Torula*, *Candida*, and *Saccharomyces* spp. as well as *Saccharomyces boulardii* CCT 4308 using coffee pulp and sugar cane molasses, respectively. It is envisaged that more instances of successful waste valorization are expected in this area of R&D in the coming years.

### 3.3. Challenges Associated with Yeast Probiotic Manufacturing

Less established entities that are interested in the use, manufacturing, and/or supply of probiotics may not have access to the skills, expertise, and infrastructure required to produce these bio-based products. These entities include but are not limited to small- and medium-enterprise farmers, start-up biotech-based companies, and the research and development community. Although these institutions and stakeholders may have a high degree of interest in probiotic development and manufacturing, they may not necessarily have the high levels of competencies in probiotic development, manufacturing, and supply in comparison to the established players [44].

In these instances, niche probiotics may be developed, or novel candidate products may be identified through collaborative R&D partnerships; however, the developmental pathway for these concepts to the commercial scale are in some instances not clear and seemed to be filled with challenges and high risk. The disadvantage of these unexploited R&D initiatives is that the value of research investments may not be realized, or the needs identified upon specific product development remain unmet [44].

Success in these instances has been attained with the creation of non-classical R&D pathways to commercialization. Feedback loops initiated within product and process development, agile manufacturing, and market and user testing coupled with the intellectual property management and regulatory frameworks (if applicable) are necessary to rapidly develop and deliver products through the value chain [44]. This also entails the specification of production performance targets, final product adherence to end-use specifications, market and financial data, and production capacity to fulfill market demand. These factors are essential in de-risking the success of the probiotic product and its adoption for use.

### 3.4. Manufacturing Considerations to Produce Yeast Probiotics

During product and process development, it is a key development area to determine baseline performance of the production process. Thereafter, ancillary development steps may be taken to further retrofit production process, especially that of cost-sensitive unit operations. Simulations and process modeling offers a useful tool, termed in silico, to enable the optimization of key process steps in the production process. With the use of this strategy, it is envisaged that significant time and expenses are saved by removing the need to conduct several actual cultivations at laboratory and pilot scales, which ultimately achieves economic impact.

**Figure 2:**
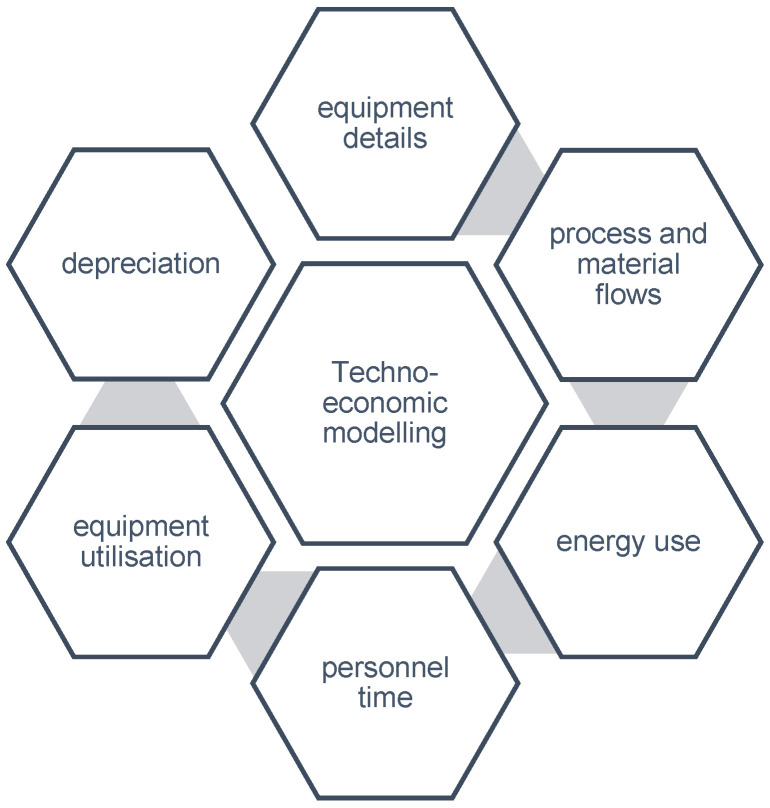
Aspects that contribute to the techno-economic feasibility of the production process.

Another important consideration for probiotic production is techno-economic modeling and assessment (Figure 2). This exercise details the technical and economic details of the process and includes the following components:

### 3.5. Location of Known Producers and Global Manufacturers of Yeast Probiotics

A key component of successfully commercializing a probiotic is the access to suitable manufacturing expertise, particularly in developing countries. Infrastructure requirements are capital intense and are limited in availability. Probiotic technologies require a production-scale pipeline that facilitates technology development from the small scale to manufacturing scale.

Large enterprises that have shown efficient production competencies have skills and infrastructure that are currently producing a wide variety of products. These production facilities are fully utilized, using tight production scheduling strategies that give them the edge of new market entrants or smaller entities that lack vertical process integration.

Biomanufacturing entities on the African continent are limited and scarcely available. The global probiotic market consists of various entities and are categorized into three tiers [56]. The tier 1 companies are listed in Table 4. As can be seen, tier 1 companies that occupy ~40 to 45% of the global market are found in the United States of America, Europe, and Japan, with most tier 2 companies also presiding in these regions.

## 4. The Use of Genetically Modified Organisms (GMO) as Probiotics

Conventionally, wild-type organisms have been applied as probiotics; however, the advances in genome editing and associated tools have unlocked the possibility of being able to engineer probiotics to deliver customized therapeutics [16]. Ma et al. [22] and other authors [3,16,22,23,24] have provided extensive reviews on the theoretical basis for probiotic gene editing technology as well as the use of these engineered probiotics for the treatment of diseases. These diseases range from inflammatory bowel disease, cancer, obesity, and diabetes, amongst others, and have been tested both in human and animal models. Interestingly, only 8% of the genetically modified organisms listed in the review were yeast probiotics, with the remaining belonging to their bacterial counterparts. It is envisaged that the advent of genome editing may impart a variety of benefits to human health especially in the treatment of specific diseases. To date, there has been significant hesitation and consumer resistance for the use of genetically modified organisms as probiotics; however, this aversion towards its use may be reduced with the progression of several clinical studies that are currently ongoing [25]. Furthermore, the use of CRISPR-Cas9 technology has been employed in demonstrating the enhanced probiotic potential of *S. boulardii* targeting various therapeutic functions outlined in Table 5.

## 5. General Routes of Administration of Yeast Probiotics

The practice of using probiotics has become widely accepted as a natural means to stimulate health for humans. Today, probiotics are used as health supplements in food, as pharmaceuticals, or chemical supplements [57]. If a probiotic is classified as pharmaceutical or drug for the treatment of a disease or disorder, stricter requirements are necessary to substantiate the claims stated by the manufacturer. It must be proven safe and effective for its intended use through clinical trials and be approved by the Food and Drug Administration (FDA) before it can be sold. Depending on the intended use of a probiotic, whether as a drug or a dietary supplement or a nutraceutical, regulatory requirements differ [58]. According to the definition provided by the FDA, a drug is an article intended for use in the diagnosis, cure, mitigation, treatment, or prevention of disease [59].

With regards to nutraceuticals, these are known as pharmaceutical alternatives that exert physiological benefits, are used to promote health, and support the structure or function of the human body [60]. Nutraceuticals are regarded as safe and less likely to have side effects; for example, probiotics are generally classified as nutraceuticals. The safety of probiotics is apparent due to the absence of toxicity in their populations. Additionally, most probiotics form part of the natural microbiome of the human host and therefore are applied back to a known environment (WHO, 2022).

In most instances, the optical concentration of active probiotic cells required to confer a positive benefit to the host is not known. What is known is that the probiotic needs to be in high enough concentration to survive some of the physiological barriers upon transit to the target site. Another important consideration is the method selected for use to deliver the probiotic to the intended site.

### 5.1. Conventional Pharmaceutical Methods Used to Administer Probiotics

#### 5.1.1. Oral Delivery Systems

**Figure 3:**
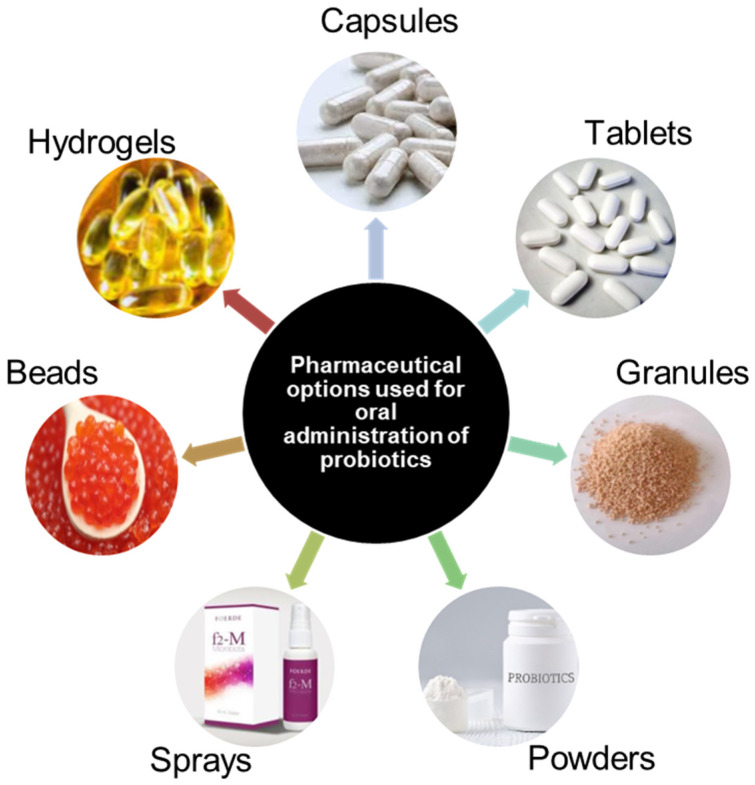
Different oral delivery systems used to administer probiotics.

The most common delivery of probiotics is oral administration. This infers that the final step in the probiotic production process is the formulation and packaging of a probiotic into a delivery system that will be able to maintain the functionality of the probiotic through the harsh gastric environment. Conventionally used delivery systems include tablets, capsules, hydrogels, granules, and others, as described in Figure 3.

Oral administration of the probiotic product is a widely accepted channel for the delivery of drugs and probiotic microorganisms in several disease and treatment applications (Table 6). Consequently, it presents the biggest challenges in administering probiotics, as the live cell preparations found in final formulated products need to ultimately survive the gastric environment in the stomach, which can reach a pH below 3 [61]. Most authors have deduced that upon the application of probiotics, when conferred in adequate amounts, these organisms are able to confer health benefits to the host. This infers that for every step in the probiotic production process, viability needs to be maintained, and cell losses need to be kept to a minimum since it is envisaged that a certain component of the probiotic population will die upon exposure to the gastric environment.

#### 5.1.2. Transdermal Delivery Systems

According to Chen et al. [26], simple and effective methods to deliver probiotics into the dermis to regulate local dermal tissue are lacking. Yeast organisms, such as *S. boulardii*, have been used to treat skin ailments, such as acne, due to its anti-inflammatory properties. However, in this instance, the probiotic product was administered dermally, as one would expect, rather than as an oral supplement. Upon ingestion, the anti-inflammatory effects of the organisms are exerted towards the skin. *S. boulardii* demonstrated the ability to produce acetic acid, which is known to exert antifungal and antimicrobial effects. This preparation, when used topically, was shown to reduce the bacterial load that causes infections or skin conditions, such as acne, rosacea, seborrheic dermatitis (scalp eczema), and eczema. In this instance, yeast by-products and not the organism itself were used as a topical treatment. All the *S. boulardii* strains tested secreted an antimicrobial agent with an inhibitory effect on *E. coli* and bioactivity against *Candida albicans* hyphae [26].

## 6. Other Probiotic Delivery Systems

It is known that the most common and effective delivery route for probiotics is orally, as the intended destination for the product is the GI tract of the host [68]. However, during the processing of pharmaceutical products in general, such as tablets, high processing temperatures may be used to obtain aesthetically pleasing final products, which may kill the probiotic microorganisms of interest. This occurrence may be applicable to many formulations that contain viable organisms and proves to be the biggest challenge that needs to be overcome in terms of final product processing.

In order to circumvent this significant challenge, fermented, also known as functional, foods are used as a delivery system and do not require high-temperature processing. Instead, indigenous microorganisms already present in the ingredient mix are activated and thereby replicate to higher concentrations. This is termed as non-conventional delivery methods despite it being consumed by several populations dating back to early human civilization.

Fermented foods derived from animals and plants play an important role in human diets, as they contain beneficial microorganisms and compounds, including organic acids, ethanol, or antimicrobial compounds [69]. These fermented foods are termed functional foods, which are foods that offer benefits that are more than their nutritional value and are divided into dairy and non-dairy options.

### 6.1. Functional Foods as a Source of Probiotics

#### 6.1.1. Dairy-Based Probiotics

In the early 1990s, Nobel Laureate Elie Metchnikoff (1845–1916), whilst working in Bulgaria, noticed how a certain population in the country had lived a longer life span than others. Upon further investigation, the researcher discovered that this population consumed fermented drinking yoghurt daily [70]. This preliminary study laid a foundation for the study of probiotics and the use of functional foods as a dietary supplement containing beneficial microorganisms containing either bacterial or yeast cultures or a combination of both. *S. boulardii*, a known yeast probiotic, has been isolated from dairy products, including milk, yoghurt, cream, cheese, and kefir.

Yeasts have shown the ability to produce enzymes that synthesize milk proteins. However, this property is only activated once lactic acid bacteria (LAB) break down the lactose present in dairy-based foods into glucose and galactose. Once this conversion is finished, both the yeasts and LAB cultures use the available sugar as a carbon source to grow and replicate [71]. Fleet, [72] added that yeasts such as *S. boulardii* should be included in products solely as a probiotic, as it offers no benefits to some dairy products, such as yoghurts; however, in cheeses, yeasts make important contributions to the process of cheese maturation. They contribute to the development of cheese flavor and texture through proteolysis, lipolysis, and the utilization of lactic acid [72,73,74].

Dairy products have been the most utilized carrier/formulation of bacterial probiotics with limited applications for yeast strains [75]. Upon assessing the literature landscape, it was found that the *Saccharomyces*, *Pichia*, *Candida*, *Meyerozyma*, *Debaryomyces*, and *Kleuveromyces* genera found in different types of fermented vegetables, cheese, and kefir [76,77,78,79]. Additionally, *Wickerhamomyces*, *Torulaspora*, *Yarrowia*, and *Metschnikowia* are the other yeast genera present in fermented fish, legumes, and meat products [75,80]. The use of dairy-based food as a means for probiotic delivery is largely affected by poor shelf-life. As a result, non-dairy-based probiotic formulations have been evaluated for their potential as an alternative solution.

#### 6.1.2. Non-Dairy-Based Probiotics

It has been found that in most instances, yeasts such as *S. boulardii* do not naturally occur in food and are instead added as a supplement. *S. boulardii* is commonly added into cereals and legumes to stabilize nutrients using its enzymes [70]. One of the nutrients that is present and broken down by the organisms is phytate or phytic acid, which is the primary storage compound for phosphorus in seeds. This compound binds to metals, rendering them insoluble and thus inaccessible as nutritional components. *S. boulardii* is added to synthesize the phytates using phytases, which in turn enhance the bioavailability and absorption of important essential minerals, such as iron, zinc, magnesium, and phosphorus [70,81,82].

Lazo-Velez et al. [70] proposed that *S. boulardii* be supplemented with cereal-based or low-water-activity foods to be used as vehicles for the addition of this probiotic yeast. Additionally, the application of *S. boulardii* has been successfully added to fermented drinks, which include beers, grain drinks, malts, and fruit/veggie juices [67,83].

## 7. Advancements in Probiotic Delivery Systems

The administration of probiotics has evolved into various methods based on the need. Typically, probiotic administration has been conducted through oral ingestion; however, new advances include the application of probiotics into the nostrils using nasal sprays, applications through the vagina, or applications as a topical application, termed transdermal applications.

An additional mechanism of oral supplementation includes the sublingual routes, whereby the probiotic is applied under the tongue, where it is absorbed rapidly. This route of administration is currently being researched, along with the advent of rectal suppositories or probiotic-based enemas. Probiotic enemas involve injecting a solution containing probiotics through your rectum and colon. This type of enema is becoming a popular alternative remedy for gut health, treatment for immune system support, and remedy for some diseases of the digestive tract.

Other avenues being explored for probiotic administration indicate that meat and meat products are emerging as potential routes when supplemented with probiotics. The most common probiotics used in meat products are mainly from the Lactobacillus and Bifidobacterium genus, and yeasts such as *S. boulardii* may be explored in future research. Probiotics in meat products have been used as bioprotective cultures against harmful and pathogenic bacteria. This probiotic effect is mainly centered on the production of bacteriocins, which aid the host [84].

## 8. Formulation Techniques Used for Yeast Probiotics

Probiotics administered orally transit through the mouth, stomach, small intestine, and the colon, where they are subjected to saliva, acidic conditions, pancreatic juices, bile acids, and digestive enzymes as well as competition with host microbiota for nutrients and adhesion sites [85]. As a result, these probiotics are shown to lose viability upon transit. This is attributed to their high sensitivity to gastrointestinal conditions, extrinsic factors such as processing techniques and storage conditions, and intrinsic factors, which include water activity, antimicrobial components, and redox potential in the product matrix [86]. As stated above, a probiotic must maintain its viable activity to confer a therapeutic effect on the host. Typically, the intended viable population is targeted to be within the range of ~1.0 × 10^6^ as a minimum up to a maximum of ~1.0 × 10^9^ CFU·mL^−1^ viable cells to be considered effective [87].

The challenges associated with the poor survival of probiotics during processing and their passage to the GIT have been studied extensively [86,88,89,90,91,92,93]. According to Serawat et al. [86], before the introduction of new technologies, there were even more challenges in the use of probiotics as starter cultures, as these preparations were used in liquid form, which was associated with a low shelf-life, a high risk of bacteriophage infections, and high production and transportation costs [86,94]. Therefore, the increased demand for the use of probiotics in food and pharmaceutical industries based on their demonstrated efficacy on health and nutritional benefits has prompted intensive research to be conducted on solving these production and viability hurdles.

### 8.1. Immobilization

Immobilization has been shown to be an effective method for the preservation of yeasts. It is typically used as a method of the entrapment of bioactive materials in protective matrices, and several reports have indicated its suitability to enhance the viability of many probiotic bacterial [95] and yeast cultures [96]. However, in some instances, yeast probiotics cells were found to be not entirely protected, as a small percentage of the immobilized material is still exposed to the external environment at the surface of the carrier and may as a consequence be deemed inefficient [95].

### 8.2. Encapsulation

Encapsulation is one of the most utilized methods for the protection of probiotics from harsh conditions and is defined as a process that involves the packaging of live probiotic cells in a food-grade material, such as polymers, proteins, and fats [21,97]. In this process, encapsulated cells are contained within the coating material, which is formed continuously around an inner core matrix [95]. Additionally, these techniques improve the bioavailability of encapsulated probiotics by facilitating a controlled release at the target site, the large intestine [98,99]. Encapsulation is categorized into two classes based on particle size, such as microencapsulation (3–800 µm) and nanoencapsulation (10–1000 nm). Since microbes are the size of a micron, microencapsulation is the only possible technique for encapsulating all probiotics, including yeasts. Microencapsulation techniques, such as extrusion, emulsion, spray drying, spray chilling, fluidized bed, freeze drying, spray-freeze drying, coacervation, and electrospraying, are currently utilized to formulate probiotics [87,99,100].

In general, encapsulation is carried out in three steps. The initial step involves the incorporation of the microbial cells into a solid or liquid matrix. The second step includes spraying and the dispersion of the solid and liquid matrix, respectively. In the third phase, the stabilization of the system is carried out either through physical (evaporation, solidification, and coalescence) or chemical (polymerization) and gelation processes.

Encapsulation was suitably demonstrated for yeast probiotic applications in a study conducted by Patarroyo et al. [27], whereby *Kluyveromyces lactis* was encapsulated in cross-linked gelatin hydrogels, which is a commercially available and relatively inexpensive material that will easily allow for industrial scale-up. The encapsulation enhanced the rigidity of the final probiotic product, as cell viability levels were enhanced by 50% under simulated GIT conditions [27].

Alginate, starch, k-carrageenan, chitosan, xanthan gum and cellulose acetate phthalate, gelatin, and milk proteins are some of the known polymers used in the encapsulation of probiotics to date [101,102,103]. The encapsulating materials are selected based on their ability to stabilize the final product, non-toxicity, and protective effect to the cells and because they possess a control release mechanism of the bioactive material in the intestinal tract [99,100]. Extrusion, spray drying, coacervation, liposomes, and emulsions are encapsulation techniques that are conventionally used in the food industry.

### 8.3. Extrusion

Although extrusion is largely employed in the encapsulation of bacterial cells, it is a low-cost, easy technique that is carried out under mild conditions and results in a high viability of encapsulated probiotics. As described by Rodrigues et al. [100], extrusion involves the use of hydrocolloid solutions containing microbial cultures. The mixture is then extruded through a nozzle in cross-linking solution, which provides an instant transition of the hydrocolloid solution to a gel, which results in the formation of beads [100]. These beads are stable at low pH levels and deform under alkaline conditions. In a study by Graff et al. [29], *S. boulardii* was encapsulated with alginate microspheres coated with chitosan by extrusion. This report revealed that only less than 1% of the non-encapsulated probiotic survived after 120 min at pH 1.1, whereas the encapsulated yeast cells remained entrapped in the microspheres. The authors further stated that exposure to pH 6.8 resulted in the release of viable yeast cells, demonstrating the effectiveness of this technique [22,29,104,105].

### 8.4. Spray Drying

In spray drying, hot gas is used to atomize a liquid product into powder instantly. It is a cost-effective and rapid microencapsulation method that results in high productivity. Spray drying is the most common process in the food industry [99,106]. However, the harsh conditions, such as high temperature, dehydration, osmotic stress, and pressure applied during the process, also pose detrimental effects to the probiotics being processed. These conditions result in the alteration of cell membrane components, such as fatty acids, proteins, and lipids, which eventual cause cell death [107]. The improvement of cell viability during spray drying has been achieved through the optimization of the process conditions, and the use of lower temperatures has been proven to be effective as a result of reduced heat damage [100,108].

### 8.5. Spray Chilling

Spray chilling is similar to spray drying, as small droplets are also produced in this technique. The matrix (formed by lipids) and the encapsulated agent are dispersed by atomization in a cold air chamber, which enables the solidification of the particles [100]. Although it is less exploited, this process is an excellent alternative for the encapsulation of probiotics due to its cost-effectiveness and applicability at the industrial scale [109,110]. In a study by Arslan-Tontul and Erbas [28], the encapsulation of *S. boulardii* by spray drying and spray chilling using Gum Arabic and b-cyclodextrin as an encapsulation material resulted in enhanced heat and survivability in the gut system [28].

### 8.6. Emulsions

During the preparation of emulsions, two immiscible liquids are dispersed in the presence of a stabilizing agent. An additional solidifying agent is used to separate the dispersed droplets. The emulsion is referred to as water-in-oil (W/O) if the dispersed phase is aqueous, whereas the opposite is named oil-in-water (O/W) or reverse phase. Simple emulsions are formed in two phases, and the addition of another phase results in double emulsions, such as water-in-oil-in-water (W/O/W) or oil-in-water-in-oil (O/W/O) [99]. This process has been widely employed in pharmaceutical and food industries to improve the solubility, activity, and stability of immiscible compounds. This system, particularly, the dispersed aqueous phase, is mostly used in the encapsulation of probiotics due to the hydrophilic properties of microbial cells. In a study by Suvarna et al. [22], effects on encapsulation by the emulsification technique were reported on four probiotic yeasts, such as *Pichia barkeri* VIT-SJSN01, *Yarrowia lipolytica* VIT-ASN04, *Wickerhamomyces anomalus* VIT-ASN01, and *Saccharomyces cerevisiae* VIT-ASN03. This resulted in enhanced survival during storage and in simulated GIT conditions [22].

### 8.7. Fluidized Bed Drying

The fluidized bed drying technology is carried out through the atomization of a coating over solid particles in suspensions. It is mainly used for coating, granulation, and drying. Fluidized bed drying is a rapid, low-cost process that has high productivity [97]. This process is attractive, as it allows for the use of various encapsulating materials, such as lipids, proteins, and polysaccharides. The particles to be encapsulated are kept in constant motion due to air flow in a heated chamber. The coating particle size is reduced, forming a solid homogenous layer [100]. The ability of this technique to protect yeast cells along with the use of Hongqu rice peptides as a microencapsulation during thermal processing was investigated by Chen et al. [111]. It was found that the drying rate and yeast viability were significant in comparison to free cells [111]. Another potential probiotic yeast, *Meyerozyma guilliemondii* Lv196, and stable granulated prototypes were reported with a 0.2% loss of viability over 15 months of storage at room temperature [112,113,114,115].

### 8.8. Supercritical Technology

Other microencapsulation techniques include supercritical technology and freeze drying. Supercritical fluids are attractive alternative solvents that describe the state of a material above its critical point at which its vapor–liquid phase equilibrium can exist. Above these conditions, the liquid–gas phase transition disappears and the properties, such as diffusion coefficient and density, continuously change with variation in pressure or temperature. Supercritical processes result in micro- or even nanoparticles with narrow size distribution and can also be used to achieve microencapsulation and surface coating of probiotics [107]. Supercritical carbon dioxide (scCO_2_) is one of the most commonly used supercritical fluids due to its environmental compatibility and low reactivity and low critical parameters. In the supercritical technique, the probiotic cells are first immobilized during the process of interpolymer complex formation in scCO_2_, and then, the probiotic microcapsule is obtained by gasifying the scCO_2_ through depressurization [116,117,118]. This technique has mainly been applied in the encapsulation of probiotic bacteria.

### 8.9. Freeze Drying

Freeze drying is one of the well-established processes in probiotic processing. The technology involves the freezing of microbial cells at an extremely low temperature and drying though sublimation under a high vacuum [107]. In comparison to spray drying, the operating conditions are less harsh and usually result in high survival rates. However, this process formation of extracellular crystals, which results in high osmotic pressure, causes cell damage. Therefore, the use of cryoprotective agents is generally applied to protect the cells. These cryoprotectants can be low-molecular-weight sugars, such as glucose, lactose, mannose, trehalose, and sorbitol, or high-molecular-weight polysaccharides and proteins [107,119]. This technique has been successfully applied in the encapsulation of the commercially available yeast probiotic, *S. boulardii*. In a study by Thomas et al. [120], *S. boulardii* was encapsulated using layers of chitosan and dextran sulphate, whereby the coated cells were subsequently frozen in liquid nitrogen before freeze drying. This resulted in enhanced viability and the permeability of the encapsulated cells [120].

### 8.10. New Advances in Probiotic Formulations

**Figure 4:**
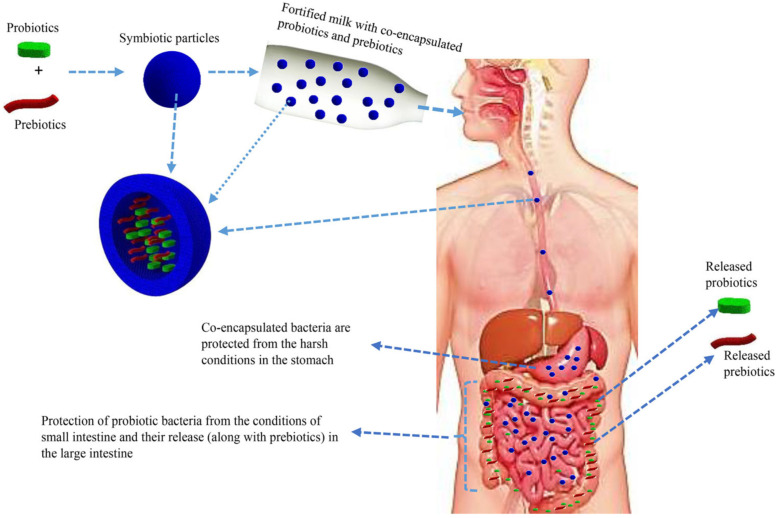
An illustration of co-encapsulated probiotics, prebiotics in a fortified milk product, and advantages offered by the technique [90].

The co-encapsulation of probiotics with prebiotics and the use of duocaps are emerging technologies applied to further enhance survivability and probiotic efficacy. Co-encapsulation improves the oral delivery of viable cells towards the target site. As reviewed by Rashidinejad et al. [90], various studies have been reported, and several polymers and polysaccharides, such as inulin, fructo-oligosaccharides, and lactulose, have been used. As illustrated in Figure 4, co-encapsulation of a probiotic with a prebiotic, enhances cell viability by providing a protective layer to the cell, while also enhancing its self-proliferation [90]. Co-encapsulated particles are referred to as synbiotics. To the best of our knowledge, there have not been any reports on the co-encapsulation of yeast probiotics, to date.

### 8.11. Emerging Trends in Products and Application Areas

When referring to probiotic products, these include functional foods, food additives and supplements, animal and/or human drugs/therapeutics, cosmetic raw materials, fermented milk, kefir, amongst others [121]. As consumers are becoming more health conscious, their search for probiotic additives has increased. In another development, due to high levels of dietary intolerance, there is also an increase in demand for non-diary-based formulations containing probiotics [122]. In developing countries, such as India, the onset of probiotic applications as a form of treatment was highlighted in 2012. Since then, there has been a wide array of probiotics containing functional foods that have been developed and been made available on the market; however, it is still increasingly difficult to attribute health claims to probiotic functionality through evidence-based clinical studies [123]. Moving away from traditional dietary supplements, there have been additional developments in the following, more specialized probiotic applications areas, detailed in Table 7.

## 9. Application of Probiotics for Preventative Health Benefits

### 9.1. Gut Microbiome Initiatives

Since the discovery of microorganisms in the 17th century, technologies and knowledge in this field have advanced rapidly, consequently resulting in microbiome mapping initiatives becoming a reality in the 21st century. Arnold et al. [35] stated that as an intrinsically multidisciplinary field, microbiome research has been able to reap the benefits of technological advancements in systems and synthetic biology, biomaterial engineering, and traditional microbiology. Prior to microbiome mapping, DNA technology and improvements thereof paved the way for advancements in whole-genome sequencing and microbial population study shifts in the human body. Further advancements have resulted in knowledge of how specific microbial compounds and activities result in health benefits, which has been a developing area of research and development [130].

The human body hosts complex microbial communities whose combined membership outnumbers our own cells by at least a factor of 10. The total number of microorganisms in the human body can reach ~100 trillion. The cells are responsible for awarding us with crucial traits, which include our reliance on them to aid in nutrition, resist pathogens, and educate our immune system [131]. In comparison to other parts of the body, the human gut has the largest number of microbes, as both the gut and skin are immensely immersed with microbiota. It is estimated that the skin has about 10^12^ cells, while the gut accounts for 10^14^ cells [132,133].

The subsequent sections will focus on the gut and skin microbiome, which interestingly share astoundingly similar characteristics, as they are highly analogous to each other, both in terms of purpose and functionality [134]. According to O’Neill et al. [135], both organs are highly innervated and vascularized, as they are both essential for immune and neuroendocrine function. The inner surface of the gut and the outer surface of the skin are both covered by epithelial cells (ECs), which have direct contact with the exogenous environment [136].

### 9.2. The Gut Microbiome

**Figure 5:**
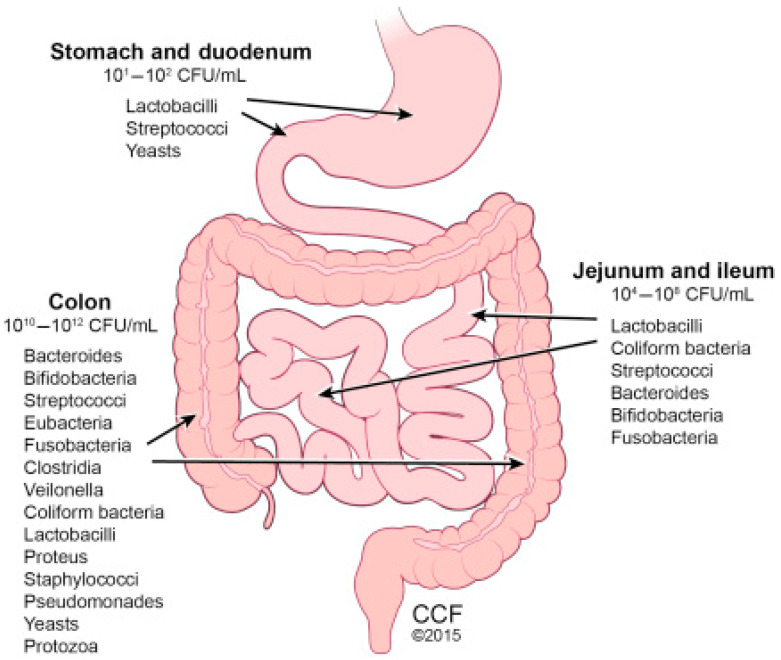
The general composition of the human gut microbiome [137].

According to Thursby and Juge [36], the human gastrointestinal (GI) tract represents one of the largest interfaces (250–400 m^2^) between the host, environmental factors, and antigens in the human body. In an average lifetime, around 60 tons of food pass through the human GI tract along with an abundance of microorganisms from the environment, which impose a huge threat on gut integrity. The digestive process starts after the ingestion of food in the mouth, where the food is grinded by teeth into smaller particles, which are then emptied into the mouth. Due to the harsh environment in the stomach, the microbial community that exists is at a low concentration of ~10^2^ cells. Once food is passed from the stomach, the contents called chyme are emptied slowly into the small intestine. In the small intestine, the duodenum, jejunum, and ileum are added; food is mixed with digestive juices from the pancreas, liver, and intestine, and the mixture is pushed forward for further digestion. In the small intestine, the microbial community can reach between 10^4^ to 10^6^ cells (Figure 5). Thereafter, all non-absorbed nutrients and waste matter that were not absorbed or used are passed into the colon, where there are between 10^12^ and 10^14^ cells (Figure 5 [137].

Microorganisms (bacteria, archaea, and eukarya) that colonize the GI tract may exert several benefits to the host through a range of physiological functions. These may include but are not limited to improving gut integrity or shaping the intestinal epithelium, harvesting energy, protecting against pathogens, and regulating host immunity. However, there is potential for these mechanisms to be disrupted as a result of an altered microbial composition known as dysbiosis [32,33,36,138,139].

Thursby and Juge [36] also added that the role of gut microbiota in human health has gained increasing attention. Several studies found that the human gut is colonized by diverse groups of bacteria species whose composition is strongly linked to every person’s individual GI health. There is also growing evidence that the administration of probiotics contributes to the microbial ecosystem, which exerts a variety of health benefits, including the prevention and/or treatment of diseases [140].

Microbial colonization of the GI tract mucosal tissue starts from infancy; these early life events have a long-standing consequence to the development of the human body and how it responds to diseases. During development from infancy, the developing microbiome is responsible for facilitating tolerance to environmental exposures or contributing to the development of diseases, including inflammatory bowel disease, allergies, and asthma. Recent studies have begun to define a critical period during early development in which the disruption of optimal host–commensal interactions can lead to persistent and in some cases irreversible defects in the development and training of specific immune subsets [33].

The role of the microorganisms that form the microbiome is to facilitate metabolism, such as breaking down indigestible complex polysaccharides into essential nutrients, such as vitamin K and B12, butyrate, and propionate [141]. The latter has been found to have a positive effect on the epithelial barrier integrity, which plays a crucial role in protecting microbiota from pathogenic microorganisms and avoiding inflammation in the gut [142]. Researchers that focus on the composition of the human microbiome have found that the most abundant genera of fungi in the human gut are *Saccharomyces*, *Malassezia*, and *Candida* in descending order of abundance [143], with 8 out of 15 genera comprising ascomycetes and Saccharomyces comprising approximately 5–65% of the observed fungi.

Saccharomyces strains have been observed up to 96.8% of samples in recent mycobiome studies [4,144,145]. Since fungi are harbored in the gut environment, it follows that some resident species might provide a symbiotic benefit to the human host. The role of the microbiome in the GI tract and its influence on human health has unlocked a significant area of interest, and further investigation, particularly the profiling of the African microbiome, is vital for further discovery in the development of niche treatment technologies for the global population.

## 10. Skin Microbiome

The skin is the largest and most external barrier of the body from the outer environment; therefore, it is considered the external interface between the body and the environment [146]. The skin is richly perfused with immune cells and heavily colonized by microbial cells, which in turn train immune cells and determine the well-being of the host. Also, it is worth noting that despite the skin covering many areas of the human body, the population and microbial concentration differs per area. It has also been found that a shift in population can also shift depending on the external environment, disease, and diet [147].

The skin epidermis, along with its appendage structures, such as sweat and sebaceous glands, provides a total skin surface of about 25 m^2^ and is one of the largest epithelial surfaces for interaction with microbes [134]. Epithelial cells cover the surfaces of the body, such as skin, airways, or the intestinal tract, and provide an important link between the outside environment and the body interior [136].

Like those in our gut, skin microorganisms have important functions in protection against invading pathogens, teaching our immune system, and the breakdown of natural products [133,148,149,150]. According to Byrd et al. [150], several skin microbiome assessment surveys have to date used amplicon sequencing; however, in recent years, major technical breakthroughs have occurred that use shotgun metagenomic sequencing. The advantage of using the latter approach is that it does not sequence specific target regions. This technique simultaneously captures all genetic material in a sample, including human, bacterial, fungal, archaeal, and viral microorganisms, providing vital information on the microbial composition.

## 11. The Use of Yeast Probiotics in Skin Applications

Further to the limited instances of the use of yeasts as probiotics employed as food supplements and/or additives, there are lesser studies that focus on the use of these organisms for skin applications. This scarcity could be because *S. boulardii*, the most-studied yeast probiotic, is mostly active in the colon and can grow at low pH levels (2.0–3.0), whereas the skin pH is 5.5. Other yeast genera, such as *Candida*, *Cryptococcus*, *Epidermophyton*, *Hortaea*, *Malassezia*, *Microsporum*, and *Trichophyton*, are well known for causing vaginal yeast infections, athlete’s foot, jock itch, ringworm, or thrush owing to their ability to penetrate tissues [151], and reports of beneficial yeasts are limited in contrast.

## 12. Case Studies Assessing the Use of Yeast Probiotics and Their Impact on the Host Microbiome

The microbiome of a healthy individual consists of balanced populations of both beneficial and harmful microorganisms [152]. These play a major role in providing the host with physiological, metabolic, and immune functions that are useful in warding off pathogens, and any imbalance results in increased levels of harmful microbes. There is a mutual relationship between the gut and human flora. The colon harbors the highest population; however, only <0.1% of these are fungi, and *Saccharomyces* and *Candida* are the dominant genera [153,154,155]. As the most commercialized probiotic yeast, *S. boulardii* is widely used in the treatment of gut-related diseases, such as Traveler’s diarrhea, AIDS-associated syndrome, irritable bowel syndrome, and Crohn’s disease. The oral administration of *S. boulardii* alone or in combination with other probiotics has been proven to induce changes in the gut microbial combinations in various clinical reports [154,156,157,158]. *S. boulardii* influences the host microbiome through the direct inhibition of pathogenic intestinal microbes and by normalizing the pH of the gastrointestinal tract; this is achieved by reducing the pathogenicity of toxic microorganisms [155].

A recent study representing the effect of the use of *S. boulardii* on the gut microbiota was reported by Yang et al. [30]. The potential of this probiotic yeast in the treatment of non-alcoholic steatohepatitis (NASH) in mice through the gut–liver axis was demonstrated. NASH is a non-alcoholic fatty liver disease associated with inflammation, damage, and the presence of excess fat in the liver. Yang et al. [30] fed a NASH-inducing diet [Methionine-choline-deficient (MCD)] to all test mice, and the control group was given a normal chow diet (NCD). Florastor^®^, a commercial product containing lyophilized *S. boulardii* CNCM I-745 as a main component, was also administered by gavage to random mice (both on the MCD diet and the control group) five days a week. After 8 weeks, the mouse fecal genomic DNA was extracted, sequenced, and analyzed. The positive effect of administering *S. boulardii* to MCD diet-fed mice was evident (summarized in Table 8) by the microbial composition presented at the family level [30]. It was concluded that this probiotic played a role in restoring the gut microbiome diversity that was reduced by the diet. Additionally, the MCD diet resulted in changes in the mycobiome, dominated by *Pichia* and *Trichosporon*. This was an indication of the robustness of these fungal genera under severe conditions in the gut [30]. Furthermore, the positive impacts of the gut mycobiota on regulating functions of other human organs, such as the brain, pancreas, liver, and kidney as well as overall host immunity towards intestinal and extraintestinal diseases, have been comprehensively reviewed by Wu et al. [159].

The co-supplementation of a multi-strain probiotic has also been shown to have even outstanding benefits. The World Health Organization stated that “mental health is critically important for everyone, everywhere” (WHO, 2022); the positive impacts of probiotics in cognitive performance were reported by Bloemendaal et al. [34]. This was determined by the increase in the population of plant-fiber-degrading bacteria that produce short-chain fatty acids, which are known for their beneficial effect on gut and brain health [34].

In another study, the benefits of the co-supplementation of bacterial (*Lacticasebacillus rhamnosus*) and fungal (*S. boulardii*) probiotics protected the gut microbiome post antibiotic administration in vitro [31]. Here, the human intestinal ecosystem was simulated using SHIME model. Three regions of the gastrointestinal tract were represented, the upper part, proximal, and distal colon. Mucin-covered mucosms were included in the proximal colon to simulate luminal mucus-associated microbiota, and the parameters in the reactors were stabilized for two weeks. The study involved two healthy human adults who consented to give fecal samples. After inoculation, baseline conditions were established, and then, a 5-day antibiotic (amoxicillin and clavulanic acid) treatment was initiated. The study was conducted in parallel, where one set was dosed with probiotics (*L. rhamnosus* and *S. boulardii*). Composition of the gut microbiota was then profiled. Although the overall population was donor-dependent, there was a clear protective impact of the yeast probiotic towards *L. rhamnosus* against antibiotics. Furthermore, the presence of each or both probiotics significantly enhanced the abundance of other *Lactobacillaceae*, *Bifidobacteriaceae*, and *Lachnospiraceae*. This demonstrated the ability of probiotics to restore, stimulate, and strengthen the composition as well as functionality of the microbial community negatively impacted using antibiotics [31].

The functionality of yeasts as probiotics is not only limited to their use as whole-cell therapeutics. A review conducted by Saber et al. [5], indicated that their metabolic by-products, such as folic acid and β-glucan, may have an effect on cancerous cells by being able to affect pathogenic bacteria; inactivate carcinogenic compounds, particularly those derived from food; improve intestinal barrier function; and modulate host immune responses, antitoxic functions, apoptosis, and anti-proliferative effects [5].

## 13. Modes of Action

**Figure 6:**
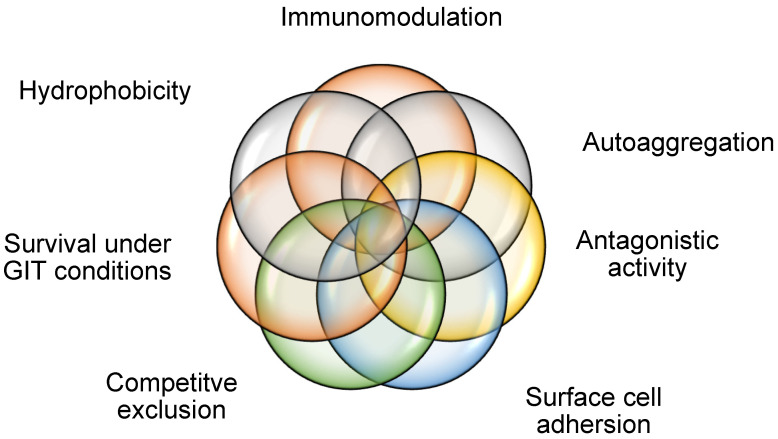
Illustrative summary of the main mechanisms of action of yeast probiotics.

General properties for yeast probiotics have been outlined in Table 3 and Figure 6 and reviewed by various authors [106,107]. These therapeutic agents offer health benefits through various mechanisms of actions, namely antagonistic activity, surface cell adherence, hydrophobicity, auto-aggregation, biofilm formation, and enhanced survival in GIT conditions.

Yeast probiotic antagonism is facilitated by various factors, including the production of bioactive compounds such as enzymes, antioxidants, antimicrobial agents, and volatile organic compounds [108]. Probiotics secrete proteases, amylases, lipases, and cellulases. These enzymes contribute to digestion, the maintenance of balance in the gut microbiome, and functions that support the intestinal barrier [108]. Additionally, chitinases and glucanases also aid in antagonistic function in yeast probiotics [109].

Another mode of action in which probiotics regulate antagonistic functions is through the competitive exclusion of pathogens for both nutrients and binding sites. This is achieved through their survival at low pH and the production of bacteriocins [109]. A well-studied antagonistic action of *S. boulardii* is against *Clostridium difficile.* This probiotic has been shown to antagonize *C. difficile* through the increased production of immunoglobulin A (IgA) and neutralization of toxin A and B, which are primary causes of diarrhea and colitis [110].

The antioxidant activity of *S. boulardii* has also been shown to play a role in biotherapeutic properties (the reduction of oxidative stress) of this probiotic. According to Abid et al. [155], this activity is attributed to the cell wall composition of this yeast consisting of vitamin B6, cinnamic acid, vanillic acid, and erythromycin [111].

Surface cell adherence is one of the key modes of actions by which the gut microbiome is maintained in the GIT [112]. This probiotic function is regulated by various surface molecules, such as flagella, pili, surface layer proteins (SLPs), capsular polysaccharide (CPS), and lipoteichoic acid lipopolysaccharide, constitute microbial-associated molecular patterns (MAMPs). Surface cell adherence is modulated by specific pattern recognition receptors [113]. Probiotics then influence the formation of the mucous layer, which provides a barrier function, hindering the adhesion and invasion of pathogens. Liu et al. [113] conducted a comprehensive review on interactions between intestinal probiotics and the gut barrier on the molecular level. Furthermore, these authors provided details on how surface layer proteins and bioactive molecules influence adhesion as a mechanism of action for probiotics [113].

Other modes of action that probiotics utilize include hydrophobicity, auto-aggregation, and biofilm formation. All these ultimately contribute to the modulation of immunity on the hosts. A summary of modes of action in which probiotics modulate immunity is illustrated in Figure 6.

## 14. Conclusionary Remarks and Future Prospects

The use of probiotics has gained significant momentum in terms of advocation for use amongst the global population. The advent of genetically engineered probiotics may be more effective, have cheaper production costs, and have higher stability and specificity for the treatment of a plethora of human ailments and disorders [3]. However, the application of these GMOs faces significant hurdles, particularly in terms of biosafety considerations upon ingestion by the host. Several clinical trials have been conducted to date; however, their effect cannot be guaranteed to achieve their intended effect, which therefore prevents effective deployment. With more in-depth understanding into the human microbiome and its relation to disease mechanisms, the safety and endorsement of engineered probiotics, both bacterial and yeast, may gain acceptance for use, particularly when conventional health strategies prove ineffective [3]. Additionally, in terms of advances in probiotic production and formulation, significant strides have been made to deliver highly efficacious probiotic treatments for the treatment of several human metabolic disorders.

## Figures and Tables

**Table 1:** Common yeast species and their probiotic traits.

Yeast Species	Probiotic Effect	Health Benefits	Applications	References
*Saccharomyces boulardii*	Antimicrobial activity, gut flora restoration, immune modulation	Treats diarrhea, prevents gut infections (e.g., *Clostridium difficile*), modulates immune response	Supplements for gut health, preventing gastrointestinal infections, and immune support	[4,18]
*Saccharomyces cerevisiae*	Glycemic control, immune modulation, antioxidant properties	Enhances metabolic health (diabetes management), supports immune function, reduces oxidative stress	Supplements for managing diabetes, cardiovascular health, and improving metabolic function	[19,20]
*Pichia kudriavzevii*	Cholesterol reduction, bile salt resistance	Lowers cholesterol, supports cardiovascular health, enhances digestive health	Supplements targeting heart health and cholesterol management	[10,21]
*Candida famata*	Antimicrobial properties, bile tolerance	May improve gut health, contributes to cholesterol management, modulates immune response	Probiotic formulations for gut and cardiovascular health	[6,9]
*Kluyveromyces lactis*	Gut adhesion, antimicrobial effects	Supports oral health (reduces oral pathogens), enhances digestive health, modulates gut flora	Oral care products, supplements for gut and dental health	[12]
*Debaryomyces hansenii*	Immune modulation, bile salt deconjugation	Boosts immune function, prevents gastrointestinal infections, supports cardiovascular health	Probiotics for immune system support and cardiovascular benefits	[6,11]
*Issatchenkia orientalis*	pH resistance, bile tolerance, pathogen inhibition	Improves digestive health, alleviates symptoms of allergies, supports gut health	Supplements for gut health, immune system balance, and allergy prevention	[10]
*Wickerhamomyces anomalus*	Biofilm formation, antimicrobial activity against pathogens	Supports oral and gut health, reduces oral pathogens (e.g., *Streptococcus mutans*), contributes to dental health	Probiotic supplements for dental health, gut health, and prevention of oral infections	[16,22]

**Table 2:** Main highlights and implications of reviewed studies.

Microorganisms Used in the Study	Highlights	Author
*Pichia fermentans*, *Pichia kudriavzevii*, *Yarrowia lipolytica*	Demonstrated yeast strains capable of cholesterol reduction, supporting their potential role in managing hypercholesterolemia.	[21]
*Saccharomyces cerevisiae*	Supplementation with *S. cerevisiae* improved glycemic indices in patients with type II diabetes, supporting its use for metabolic health.	[19]
*Saccharomyces cerevisiae* and *Lactobacillus rhamnosus*	Probiotics inhibited and reduced *Gardnerella vaginalis* biofilms in mice, demonstrating potential in treating bacterial vaginosis.	[23]
Engineered probiotics	Engineered probiotics show potential for customized therapeutic applications in treating diseases like inflammatory bowel disease, cancer, and obesity. Significant regulatory and biosafety concerns remain.	[3]
*Meyerozyma caribbica* 9D	Yeasts isolated from pineapple-improved sensory properties of fermented beverages while reducing ethanol content, showcasing probiotic potential in beverages.	[24]
*Kluyveromyces*, *Torula*, *Candida*, *Saccharomyces* spp.	Demonstrated cost-effective yeast probiotic production using agro-industrial residues like coffee pulp, promoting sustainable waste valorization.	[25]
*Saccharomyces boulardii*	*S. boulardii* was successfully cultivated using sugarcane molasses, reducing production costs for large-scale probiotic production.	[26]
*Saccharomyces boulardii*	Novel transdermal delivery of live probiotics using dissolvable microneedle patches, presenting a new method for direct skin administration.	[26]
*Kluyveromyces lactis*	Encapsulation in cross-linked gelatine hydrogels enhanced viability in gastrointestinal conditions, highlighting effective encapsulation techniques.	[27]
*Saccharomyces boulardii*	Encapsulation by spray drying and spray chilling using gum Arabic improved heat resistance and survival in the gastrointestinal tract.	[28]
*Saccharomyces boulardii*	Encapsulation of *S. boulardii* with alginate microspheres coated with chitosan enhanced survival in acidic conditions, allowing for controlled release in the intestines.	[29]
*Pichia barkeri*, *Yarrowia lipolytica*, *Wickerhamomyces anomalus*, *S. cerevisiae*	Emulsification encapsulation improved survival of probiotic yeasts during storage and simulated gastrointestinal conditions.	[22]
*Saccharomyces cerevisiae*	Fluidized bed drying of encapsulated yeast enhanced viability during thermal processing and storage, showcasing a promising technique for industrial-scale production.	[26]
Yeast metabolic by-products	Yeast by-products such as folic acid and β-glucan exhibit anti-carcinogenic properties, modulate immune responses, and improve intestinal health by affecting pathogenic bacteria.	[5]
*Saccharomyces boulardii*	*S. boulardii* restores gut microbiome diversity, ameliorates non-alcoholic steatohepatitis (NASH), and shows promise in treating liver diseases via the gut-liver axis.	[30]
*Saccharomyces cerevisiae* and *Lactobacillus rhamnosus*	Co-supplementation of bacterial and yeast probiotics protects the gut microbiome after antibiotic administration, enhancing microbial restoration and functionality.	[31]
Gut microbiota	The role of gut microbiota in resisting pathogenic bacteria through competitive exclusion and immune modulation highlights probiotic potential in pathogen control.	[32]
Early-life microbiome	Early microbiome colonization in infants is crucial for long-term immune function, emphasizing the importance of early probiotic interventions for health.	[33]
Multi-strain probiotics	Co-supplementation with bacterial probiotics improved cognitive performance and gut health, highlighting the mental health benefits of probiotics.	[34]
Gut microbiome research	Technological advancements in microbiome mapping and microbial genome sequencing are driving the development of microbiome-based health interventions.	[35]
Gut microbiome health	Diverse bacterial species in the human gut are strongly linked to individual gastrointestinal health, and probiotic supplementation can help maintain microbial balance.	[36]

**Table 3:** Organism properties used to assess the potential of a yeast for use as a probiotic.

Characteristic	Rationale	Reference
Hydrophobicity	For an organism to show functionality as a probiotic, it needs to display hydrophobicity. The organism of interest needs to demonstrate its ability to adhere/interact with the mucus present within the GIT to confer the probiotic effect.	[37]
Auto-aggregation	This is a characteristic wherein cells are able to self-aggregate and adhere to the mucus/mucosal lining in order to form a biofilm. A desirable level of auto-aggregation is ~30 to 60%.	[38]
Biofilm formation	To show the ability of cells to adhere to each other and the host lining.	[39]
Adherence ability	To assess the ability of the probiotic cell to adhere to the mucosal lining and confer a probiotic.	[40]
Survival	To assess the organism’s ability to survive exposure to low pHs (gastric conditions) and the presence of bile salts (0.3%).	[41]
Antibiotic resistance	In the instances of yeasts intended for use as a probiotic, antibiotic resistance using a disc assay method will infer information pertaining to the ability of the organism to demonstrate antibiotic resistance.	[42]
Antimicrobial activity	To assess the yeast’s ability to demonstrate anti-microbial activity, which is pertinent for the treatment of pathogens.	[20]

**Table 4:** Major global producers (tier 1) of probiotics [56].

Name	Country
ADM PROTEXIN Ltd.	Somerset, NJ, USA
Abbott	Chicago, IL, USA
Asahi Group Holdings Ltd.	Tokyo, Japan
Chobani LLC	New York, NY, USA
Chr. Hansen Inc.	Hoersholm, Denmark
DSM	Heerlen, The Netherlands
Danone Inc., IFF	Paris, France
Kerry Group PLC	Tralee, Ireland
Estee Lauder Inc.	New York, NY, USA
Morinaga Milk Industry Co., Ltd.	Tokyo, Japan
NESTLÉ S.A	Vevey, Switzerland
Yakult Honsha Co., Ltd.	Tokyo, Japan

**Table 5:** Examples of genetically modified probiotics for improved therapeutic properties.

Probiotic Microorganism	Genetic Engineering Tool	Benefit	Author
*S. boulardii* ATCC-MYA796	CRISPR-Cas9	Expression of a *Clostridioides difficile* Toxin A neutralizing	[26]
		Expansion of probiotic habitat in the intestines	[27]
		Secretion of human lysozyme	[28]
*S. boulardii* ATCC MYA-797		Tuneable transactivation system for various potential therapeutic functions	[29]
*S. boulardii* CNCM I-745		Enhanced probiotic potential via secretion of the antimicrobial peptide leucocin C against *L. monocytogenes*	[5]
*S. boulardii*	Directed evolution and CRISPR-Cas9	Mediation of inflammatory bowel disease by expression of a human purinergic receptor (enhanced eATP sensivity)	[30]
*S. boulardii* CNCM I-745	CRISPR-Cas9	Virulence gene (heme-oxygenase-1 (HMX1) deletion with enhanced immunity to opportunistic infection	[31]
*S. boulardii*-PLI1	Classical gene cloning	Anti-obesity probiotic expression of a pancreatic lipase inhibitor	[32]

**Table 6:** Some of the benefits that are offered from the oral administrations of probiotics and addition in food products.

Benefits	Organisms of Interest	Reference
Inhibition of Cd absorption Protection of the intestinal barrier—by alleviation of Cd-induced oxidative stress	*L. plantarum*	[62]
Enhancement of antimicrobial activity	*L. paracasei* and *L. casei*	[63]
Reduction of hypertension effects	*S. cerevisiae*	[64]
Modification of the fecal resistome during *Helocobacter pylori* treatment—reduction of antibiotic resistance	*S. boulardii*	[65]
Potential in removal of toxins	*S. cerevisiae* W13 and *S. boulardii* ATCC MYA-796	[66]
Improvement of glycemic indices in type II diabetic patients	*S. cerevisiae*	[19]
Inhibition and reduction of *Gardnerella vaginalis* biofilms in mice	*S. cerevisiae* CNCM I-3856 and *L. rhamnosus* ATCC 53103	[23]
Cholesterol reduction	*Pichia fermentans* BY5 *Pichia kudriavzevii* BY10 *Pichia kudriavzevii* BY15 *Yarrowia lipolytica HY4*	[21]
Better sensory properties with lower ethanol content	*Meyerozyma caribbica 9D*	[24]
Production of alcohol-free and low-alcohol products	*S. boulardii*	[67]

**Table 7:** Emerging trends for probiotic use.

Application Area	Principle	Authors
Microbiome tailoring	Due to the link found between host microbiome and host response to disease, the use of probiotics as a complementary treatment has demonstrated that there are links between host dysbiosis and disease progression, such as cardiocvascular and cardiometabolic diseases. By increasing microbial gut diversity to circumvent the deficiencies, it offers a tailored personalized approach to personalized medicine.	[124]
Tailored/precision formulations	Precision probiotics are being investigated as a mechanism to rectify dysbiosis; as a form of treatment, precision probiotics are being investigated for use to restore healthy microbiome balance by using specialized tools like machine learning.	[125]
Psychobiotics	This term refers to probiotics that influences the gut–brain axis and have found applications in regulating/influencing the functioning of the nervous system in conjunction with the immune system.	[126]
Immune modulation	Probiotics and postbiotics have been found to modulate the immune system, both positively and negatively, depending on the health status of the host. In-depth studies of pro- and postbiotics are being conducted to understand their involvement in the host immunomodulatory activities.	[127]
Metabolic health/lifestyle related diseases/disorders	The composition of the gut microbiota has been shown to be an important determinant of metabolic disorders, such as obesity and type 2 diabetes. To circumvent these event, genetically modified organisms have been shown to have successful application in restoration of the gut microbiome. Synbiotics and postbiotics have been shown to regulate the gut microbiome and hence have potential for use in targeting metabolic diseases, such as obesity and type 2 and gestational diabetes.	[128]
Allergies and autoimmune diseases	Gut dysbiosis has also been found to have links that have led to conditions such as eczema, allergies, asthma, and other autoimmune diseases. In these applications, gastrointestinal immune system and probiotic cross-talk have been shown to have a direct relevance in circumventing these chronic conditions.	[129]

**Table 8:** Dominating microbial families in mice fed with normal chow diet, MCD only, and MCD and *S. boulardii* at family level.

NCD	MCD	MCD Plus *S. boulardii*
*Muribaculaceae*	*Akkermansiaceae*	*Lachnospiraceae*
*Ruminococcaeceae*	*Erysipelotrichaceae*	*Atopobiaceae*
*Lactobacillaceae*	*Tannerellaceae*	*Ruminococcaceae*

## Data Availability

Data is contained within the article. The original contributions presented in this study are included in the article. Further inquiries can be directed to the corresponding author.
